# Authentication of duck blood tofu binary and ternary adulterated with cow and pig blood-based gel using Fourier transform near-infrared coupled with fast chemometrics

**DOI:** 10.3389/fnut.2022.935099

**Published:** 2022-10-26

**Authors:** Fangkai Han, Li Ming, Joshua H. Aheto, Marwan M. A. Rashed, Xiaorui Zhang, Xingyi Huang

**Affiliations:** ^1^School of Biological and Food Engineering, Suzhou University, Suzhou, Anhui, China; ^2^School of Food and Biological Engineering, Jiangsu University, Zhenjiang, Jiangsu, China

**Keywords:** animal blood food, food fraud, FT-NIR, fast chemometrics, rapid detection

## Abstract

This work aims to investigate a feasible and practical technique for the authentication of edible animal blood food (EABF) using Fourier transform near-infrared (FT-NIR) coupled with fast chemometrics. A total of 540 samples were used, including raw duck blood tofu (DBT), cow blood-based gel (CBG), pig blood-based gel (PBG), and DBT binary and ternary adulterated with CBG and PBG. The protein, fat, total sugar, and 16 kinds of amino acids were measured to validate the difference in basic organic matters among EABFs according to species. Fisher linear discriminate analysis (Fisher LDA) and extreme learning machine (ELM) were implemented comparatively to identify the adulterated EABF. To predict adulteration levels, four extreme learning machine regression (ELMR) models were constructed and optimized. Results showed that, by analyzing 27 crucial spectral variables, the ELM model provides higher accuracy of 93.89% than Fisher LDA for the independent samples. All the correlation coefficients of the optimized ELMR models’ training and prediction sets were better than 0.94, the root mean square errors were all less than 3.5%, and the residual prediction deviation and the range error ratios were all higher than 4.0 and 12.0, respectively. In conclusion, the FT-NIR paired with ELM have great potential in authenticating the EABF. This work presents amino acids content in EABFs for the first time and built tracing models for rapid authentication of DBT, which can be used to manage the EABF market, thereby preventing illegal adulteration and unfair competition.

## Introduction

Animal blood is an excellent source of high-biological value proteins, essential minerals, and vitamins (1). The global consensus has concluded that using animal blood in foods helps in reducing the loss of valuable nutrition sources and prevents environmental pollution in the meat industry. Compared to advanced biological treatments, such as protein extraction from animal blood *via* membrane technology (2), the high-cost animal blood food, known as “blood tofu,” obtained through simple thermal processes, is widely distributed in supermarkets and restaurants in China. There are, however, differences in the types of animal blood that are suitable for making blood tofu. Compared to other commonly consumed animal blood, duck blood was more delicate and delicious (3). Therefore, in China’s domestic market, the price of duck blood is usually higher than that of cow blood or pork blood (4).

Although only a few scientific papers have addressed the issue of animal blood adulteration, it is clear from media reports and administrative punishment cases that the high-economic value of duck blood tofu (DBT) being adulterated or replaced with other low-cost animal blood remains a serious issue (3). Food frauds result in insignificant economic losses for businesses and governments, destroying brands and devaluing the market value of affected products (5). Moreover, with the advancement of novel food processing technology, it is becoming increasingly difficult to discern the authenticity of DBT by its appearances or textures. Therefore, there is an urgent need for an effective and practical technique for determining the DBT’s authenticity to promote fair trade and protect consumers’ rights.

Until now, DNA-based technology has demonstrated a high potential for determining the authenticity of animal blood. Sasimanas Unajak and co-workers developed an interspecies-specific multiplex-PCR assay using dried blood as an alternative DNA source to classify four commercial animal species: chicken, pig, cow, and crocodile (6). Yasser Said El-Sayed and co-authors researched DNA markers for detecting and discriminating human, cattle, buffalo, horse, sheep, pig, dog, cat, and chicken blood samples using a species-specific PCR method (7). Although the DNA-based technique is accurate, it suffers from several setbacks, such as DNA degradation and insufficient quantitative analysis (3). Gregory McLaughlin’s work showed that Raman spectroscopy could be effectively applied as a non-destructive technique for differentiating human blood stains from abroad range of animal blood stains, including cow, pig, chicken, etc. (8). Raman spectra provide information about the laser scattering characteristics of food surface substances. The detection depth is a vital obstacle to its practical application, as different depths of the DBT may contain varying amounts of adulteration. Recently, species-specific peptide markers of several animal blood samples were discovered and investigated for species identification using ultraperformance liquid chromatography in combination with the triple quadrupole mass spectrometry technique (3). However, this technique is a problematic practice due to the cumbersome measurement procedures, false positives due to sample contamination, and other drawbacks (4).

Fourier transform near-infrared spectroscopy is a promising green analytical technique presented with the following characteristics: rapid, convenient, non-destructive, and reliable (9). It has been widely used to develop methods for the authentication of animal source foods (10), with a particular emphasis on meat adulteration, for example, detection of pork adulteration in veal products (11), analysis of the minced lamb and beef fraud (12), chicken meat authenticity (13), quantitative detection of binary and ternary adulteration of minced beef with pork and duck meat (14), quantification of pork meat in other meats (15), and quantification of beef, pork, and chicken in ground meat (16), to name but a few. To our knowledge, however, the utilization of FT-NIR to test the authenticity of animal blood food has not been reported.

Hence, the present work attempts to develop a practical technique for the authentication of the DBT binary and ternary adulterated with cow and pig blood-based gel using FT-NIR with fast chemometrics models constructed, thereby preventing illegal adulteration and unfair competition.

## Materials and methods

### Samples prepared

Commercialized raw DBT was obtained from Henan Huaying Agricultural Development Co. Ltd. (the largest stockholder of the Chinese duck industry). Fresh cow and pig blood were purchased from a local slaughterhouse in Suzhou, China. After natural sedimentation, the cow and pig blood were sterilized for 40 min at 100°C to prepare blood gels, namely cow blood-based gel (CBG) and pig blood-based gel (PBG). All samples were homogenized and then frozen and kept at a temperature of -20°C for measurements.

A total of 540 samples were prepared and used, including 30 samples each of raw DBT, pure CBG, and PBG; 300 samples for adulterated binary DBT samples, in which the DBT was separately mixed with the CBG and PBG in a range of 10–50% by weight at 10% steps, and 30 samples on each adulteration level; 150 samples for the ternary adulterated DBT samples, in which the DBT was together mixed with CBG and PBG, in range of 10–50% by weight at 10% increments with equal amounts of CBG and PBG, and 30 samples on each adulteration level. The samples prepared and used are summarized in Table 1.

**TABLE 1:** Samples prepared and utilized.

	Adulteration levels	Sample sizes
Raw DBT	0%	30
Pure CBG	–	30
Pure PBG	–	30
DBT mixed with CBG	10%, 20%, 30%, 40%, 50%	5 × 30 = 150
DBT mixed with PBG	10%, 20%, 30%, 40%, 50%	5 × 30 = 150
DBT mixed with CBG and PBG	10% (5% CBG + 5% PBG)	30
	20% (10% CBG + 10% PBG)	30
	30% (15% CBG + 15% PBG)	30
	40% (20% CBG + 20% PBG)	30
	50% (25% CBG + 25% PBG)	30

DBT, Duck blood tofu; CBG, Cow blood-based gel; PBG, Pig blood-based gel.

### General organic matters analysis

The Kjeldahl method (GB 5009.5-2016) was used for the crude protein analysis; the Soxhlet extractor method (GB 5009.6-2016) was utilized to determine fat content in EABF samples; the Phenol-Sulfate spectrophotometry (GB/T 9695.31-2008) was applied for total sugar analysis, and the ion-exchange chromatography with post-column derivatization of ninhydrin (GB 5009.124-2016) was performed for amino acids analysis.

### Fourier transform near-infrared measurements

The Antaris II Near Infrared Spectrophotometer (ThermoElectron Company, USA) was employed to research the FT-NIR diffuse reflectance spectroscopy analysis. Due to the optical fiber used, it is possible to separate the spectrometer from the measurement location over several meters. Thus, industrial installations with a high degree of flexibility and complete automation are possible (11). The sample was collected separately into a standard cup and scanned three times with a spectral resolution of 8.0 cm^–1^ at different points. Each spectrum consisted of an average of 32 scans ranging from 4,000 to 10,000 cm^–1^, resulting in a total of 1,557 variables for each sample (17). Chemometrics was performed using the average of the three spectra collected from one sample (18).

### Chemometrics and software

Chemometric models were constructed and optimized for authentication of the animal blood food by analysis of FT-NIR datasets.

The first derivative (1st Der), second derivative (2nd Der), centralization, standard normal variate transform (SNV), and multivariate scattering correction (MSC) (19) separately paired with stepwise discriminant analysis (SWDA) (20) were used in comparison for spectra denoising and screening of the crucial spectral wavenumbers to identify the adulterated DBT.

Then, Fisher linear discriminant analysis (Fisher LDA) and non-linear algorithm, namely extreme learning machine (ELM) were utilized comparatively for modeling to identify adulterated DBT mixtures. ELM is selected because of its extremely fast learning speed, satisfactory generalization performance, and simple neural network structure (10). ELM theory for tasks of recognition and regression can be found in the literature by Pro. Guangbin Huang (21, 22).

The Fisher LDA and ELM models were evaluated for their performance in identifying DBT binary and ternary mixtures adulterated with cow and pig blood-based gels shown in Eq. (1).


(1) $$R=\frac{N_{1}}{N_{2}}\times 100\%$$

Where *R* means the prediction accuracy (%) of the training or test set; *N*_1_ means the number of correctly classified samples; *N*_2_ means the number of all samples in the training or test set.

According to the conventional detection procedures of FT-NIR for food adulteration, when the identification task of adulterated blood food is completed, the next step is to quantitatively predict the adulteration level. Similarly, the 1st Der, 2nd Der, centralization, SNV, and MSC separately paired with competitive adaptive reweighted sampling methods (CARS) (23) were used in comparison for spectra denoising and screening of the crucial spectral wavenumbers to predict the adulteration level. Extreme learning machine regression (ELMR) models were constructed and optimized for the task of predicting adulteration levels.

The root mean square error (*RMSE*) in the training set (*RMSE*_*t*_) and prediction set (*RMSE*_*p*_), the correlation coefficients (*r*) in training set (*r*_*t*_), prediction set (*r*_*p*_), the residual prediction deviation (*RPD*), and the range error ratio (*RER*) were used to evaluate ELM regression models’ performance. The *RMSE*, *r*, *RPD*, and *RER* were calculated with the following formulas:


(2) $$R\,M\,S\,E=\sqrt{\frac{\sum_{i=1}^{n}{\left(y_{i}-\overset{\wedge }{\underset{i}{y}}\right)}^{2}}{n}}$$

where *n* means the sample size of the training or prediction set, *y*_*i*_ means the actual adulteration level of the *i*th sample, and $\overset{\wedge }{\underset{i}{y}}$ means the predicted adulteration level of the *i*th sample (24).


(3) $$\text{r}=\sqrt{1-\frac{\sum_{i=1}^{n}{\left(\overset{\wedge }{\underset{i}{y}}-y_{i}\right)}^{2}}{\sum_{i=1}^{n}{\left(\underset{i}{y}-\overset{\_}{y}\right)}^{2}}}$$

where, $\bar{y}$ means the average value of the actual adulteration levels of the samples in the training or prediction set (24).


(4) $$R\,P\,D=\frac{\mathrm{Std}.}{R\,M\,S\,E_{\text{p}}}$$

where *Std.* means the standard deviation of the reference values for the training set samples. An *RPD* above 3 is considered satisfactory; a value of 5 or higher indicates that the established model can be used for quality control. Prediction models with an *RPD* value of 2–3 are considered to perform well enough for fast screening analysis (25).


(5) $$R\,E\,R=\frac{M\,\text{ax}-M\,i\,n}{R\,M\,S\,E_{t}}$$

where, *Max* and *Min* mean the maximum and the minimum values observed in the reference data (26), e.g., the adulteration levels. *RER* above 10 is roughly an indicator of a model with good predictive ability (27).

All algorithms were implemented in Matlab Version 7.14 (Mathworks, Natick, USA) with windows 10.

## Results and discussions

### General organic matters analysis results

Protein, fat, and total sugars were determined to validate the difference in basic organic matters among raw DBT, pure CBG, and PBG used in this study. Results are shown in Table 2. It was found that the fat content in DBT and PBG was significantly lower than that in CBG; the total sugars content in DBT and CBG was similar, but both significantly lower than that in PBG; the protein content in PBG was the highest, while the protein content in CBG was the lowest.

**TABLE 2:** Protein, fat, total sugars, and 16 different amino acids content (g/100 g) in the raw duck blood tofu, cow, and pig blood-based gels used.

		Duck blood tofu	Cow blood-based gel	Pig blood-based gel
Protein		27.7 ± 0.507^b^	26.7 ± 0.294^c^	28.8 ± 0.438^a^
Fat		0.0391 ± 0.016^b^	0.126 ± 0.007^a^	0.0438 ± 0.002^b^
Total sugars		1.42 ± 0.164^b^	1.31 ± 0.093^b^	1.96 ± 0.513^a^
Amino acids	Aspartic acid	2.88 ± 0.059^a^	2.62 ± 0.061^b^	2.91 ± 0.051^a^
	Threonine	1.54 ± 0.013^a^	1.37 ± 0.026^b^	0.871 ± 0.016^c^
	Serine	1.36 ± 0.012^b^	1.47 ± 0.028^a^	1.20 ± 0.022^c^
	Glutamic acid	3.35 ± 0.03^a^	2.51 ± 0.051^b^	2.20 ± 0.042^c^
	Proline	1.24 ± 0.049^a^	0.993 ± 0.019^b^	0.926 ± 0.035^c^
	Glycine	1.22 ± 0.009^a^	1.08 ± 0.021^b^	1.13 ± 0.021^c^
	Alanine	2.67 ± 0.018^a^	1.79 ± 0.034^b^	1.89 ± 0.035^c^
	Valine	1.74 ± 0.007^b^	1.65 ± 0.043^c^	1.83 ± 0.036^a^
	Methionine	0.385 ± 0.006^a^	0.203 ± 0.009^b^	0.135 ± 0.002^c^
	Isoleucine	0.874 ± 0.011^a^	0.340 ± 0.008^b^	0.176 ± 0.006^c^
	Leucine	3.13 ± 0.033^a^	2.79 ± 0.055^b^	3.09 ± 0.058^a^
	Tyrosine	1.05 ± 0.023^a^	0.762 ± 0.023^b^	0.578 ± 0.008^c^
	Phenylalanine	2.01 ± 0.026^a^	1.68 ± 0.021^b^	1.63 ± 0.040^b^
	Histidine	1.72 ± 0.018^a^	1.42 ± 0.023^c^	1.60 ± 0.028^b^
	Lysine	2.75 ± 0.027^a^	2.28 ± 0.026^b^	2.21 ± 0.031^c^
	Arginine	1.64 ± 0.014^a^	1.13 ± 0.025^b^	1.02 ± 0.023^c^

Results are expressed as mean values ± standard deviation using three significant digits, *n* = 9 for protein, lipids, total sugars content analysis; *n* = 6 for amino acids content measurement. Values in the same line with different superscripts were significantly different (*P* < 0.05). Superscript a stands for the maximum value, c stands for the minimum value, and b stands between a and c.

It could also be found that protein is the major organic component in animal blood food used. As shown in Table 2, 16 different kinds of amino acid contents in samples were measured to explore the differences in protein composition. The aspartic acid and leucine content in DBT and PBG were significantly higher than these two indicators in CBG; the phenylalanine content in CBG and PBG was significantly less than that in DBT. The remaining 13 kinds of amino acids differed significantly among DBT, CBG, and PBG. It was found that the content of threonine, glutamic acid, proline, glycine, alanine, methionine, isoleucine, tyrosine, lysine, and arginine in DBT and PBG were the highest and the least, respectively. The serine content in CBG was significantly higher than that in DBT or PBG, while the serine content in PBG was the lowest. The valine content in the animal blood food samples increased in the following order: CBG, DBT, and PBG; and Histidine content was CBG, PBG, and DBT in turn.

### Identification of the adulterated duck blood tofu

**FIGURE 1:**
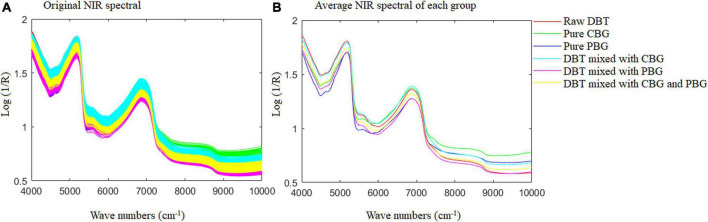
The original NIR spectral for the samples prepared and used. DBT, Duck blood tofu; CBG, cow blood-based gel; PBG, pig blood gel.

Fisher LDA and ELM were utilized for modeling to identify the adulterated DBT. All 540 samples were divided into six categories: (1) Raw DBT, (2) Pure CBG, (3) Pure PBG, (4) DBT mixed with CBG, (5) DBT mixed with PBG, and (6) DBT ternary mixed with CBG and PBG, whose original FT-NIR spectral data are shown in Figure 1.

During modeling, one-third of the samples in each group were selected as the prediction set using the Kennard-Stone algorithm (28), and the remaining samples were utilized as the training set. The adopted Kennard-Stone algorithm ensures that the samples of training and test sets remain unchanged in the modeling process under the conditions of different spectral preprocessing methods and selected key variables, which is helpful to improve the stability of the model.

The 1st Der, 2nd Der, centralization, SNV, and MSC separately paired with the SWDA were performed and compared for spectra denoising and selecting the crucial spectral wavenumber. Afterward, the principal component analysis (PCA) was used to reduce the dimension and decorrelate the variables selected. The top three principal components (PCs) of the selected spectral variables with different spectral pretreatment techniques were utilized because of their cumulative contribution rates were all over 99.0%.

As for the identification of the adulterated DBT, the results showed that the optimal Fisher LDA model obtained by selecting 27 critical spectral wavenumbers through SWDA and MSC was the best spectral preprocessing technique. The discriminate functions (DFs) are used as follows:


(6) D⁢F⁢1=-0.146*P⁢C⁢1-0.9791*P⁢C⁢2-0.1416*P⁢C⁢3;

(7) D⁢F⁢2=-0.2469*P⁢C⁢1+0.1989*P⁢C⁢2-0.9484*P⁢C⁢3;

(8) D⁢F⁢3=0.1181*P⁢C⁢1-0.0423*P⁢C⁢2-0.9921*P⁢C⁢3.

**FIGURE 2:**
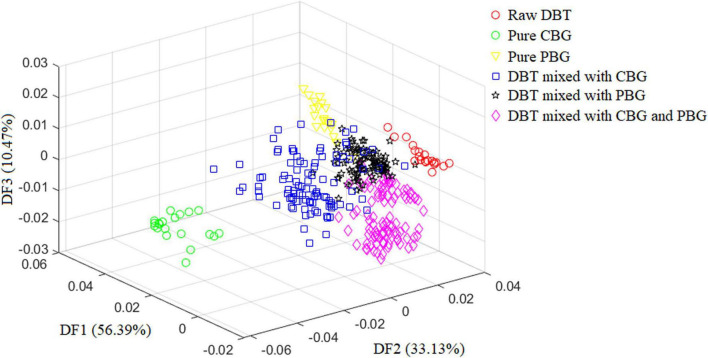
The scatter diagram of the discriminate scores of the training set samples for the optimal Fisher LDA model. DBT, Duck blood tofu; CBG, cow blood-based gel; PBG, pig blood gel.

**FIGURE 3:**
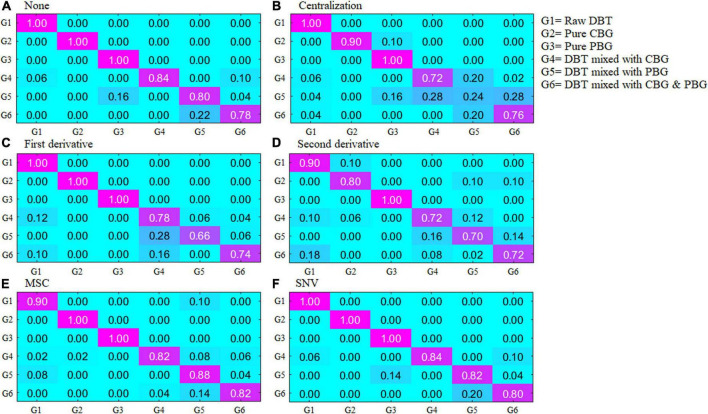
The confusion matrixes of the training set of the Fisher LDA. DBT, Duck blood tofu; CBG, cow blood-based gel; PBG, pig blood gel.

Figure 2 depicts the scatter diagram of the discriminate scores of the training set samples of the optimal Fisher LDA model. Herein, 27 samples were misclassified, and the prediction accuracy was 92.5%. In terms of the test set, 25 samples were misclassified, giving a prediction accuracy of 86.11%. The following samples were incorrectly classified: one sample of the DBT was misclassified as DBT mixed with PBG; nine samples of DBT mixed with CBG were misclassified, including four samples as DBT mixed with PBG, three samples as DBT ternary mixed with cow and pig blood-based gels, one sample as DBT, and one sample as CBG; six samples from the group of DBT mixed with PBG were misclassified, there were four samples misclassified as DBT, and two samples as DBT ternary mixed with cow and pig blood-based gels, respectively; nine samples from the group of DBT ternary mixed with cow and pig blood-based gels were misclassified, including seven samples misclassified as DBT mixed with PBG, and two samples as DBT mixed with CBG. Figure 3 shows the confusion matrixes of the training set of the Fisher LDA. Table 3 summarizes the results of all Fisher LDA models with various spectral denoising techniques and crucial variables selected using SWDA.

**TABLE 3:** Identification rates of the Fisher LDA models to classify the adulterated duck blood tofu with different spectra preprocess methods and wavenumbers selected.

Methods	No. of the wavenumber used	Training set (%)	Test set (%)
None	12	91.94	83.89
Centralization	10	67.78	63.89
First derivative	56	82.5	77.22
Second derivative	66	84.17	74.44
**MSC**	**27**	**92.5**	**86.11**
SNV	12	92.78	85.00

Bold digits represent the optimal performance.

Extreme learning machine model was also constructed using the same training and test sets with the optimal Fisher LDA model. The ELM theory states that the number of hidden neurons and the activation function of the hidden layers have a significant impact on the ELM’s performance (29). Hence, three functions were utilized in comparison as the activation function for the hidden layers during ELM modeling, as depicted in the following formulas:


(9) $$\mathrm{Sigmoidal}\,\mathrm{function}:S\,\left(x\right)=\frac{1}{1+e^{-x}}$$

(10) Sine⁢function:S⁢(x)=sin⁢(x)

(11) Hardlimfunction:S(x)={0x≤0.1⁢x>0;

Generally, the optimal number of hidden neurons can be obtained *via* the cut-and-trial method. However, the range of the optimal hidden neurons number could be obtained with the aid of the following empirical formula (30),


(12) $$N_{\text{h}}=\frac{N_{S}}{\left(\alpha *\left(N_{i}+N_{0}\right)\right)}$$

where, *N*_*h*_ means the number of hidden neurons, *N*_*s*_ means the sample size of the training set, *N*_*i*_ means the number of the input neurons, and *N*_0_ means the number of output neurons, α = [2, 10].

For this work, *N*_*s*_ = 360, *N_*i*_* = 3, and *N*_0_ = 1. Hence, the *N_*h*_* = [9, 45], as the strategy of cut-and-trial used, the optimal number of the hidden neurons was set at a range of [1, 50].

**FIGURE 4:**
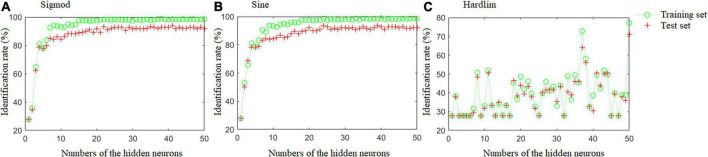
The results of the ELM models with different activation functions and numbers of hidden neurons.

Figure 4 depicts the results of the ELM models with different activation functions and hidden neuron counts. The optimal ELM model was obtained when the number of hidden neurons was 41 and the Sine activation function was used, which offered the highest identification rates of 98.61 and 93.89% in the training and test sets, respectively. Details of the misclassified samples in the test set are as follows: one DBT sample was misclassified as DBT adulterated with CBG; five samples from the group of DBT adulterated with CBG were misclassified, including three samples as DBT ternary adulterated with cow and pig blood-based gels, and two samples as DBT adulterated with PBG; two samples from the group of the DBT adulterated with PBG were misclassified as DBT adulterated with CBG; three samples from the group of the DBT ternary adulterated with cow and pig blood-based gels were misclassified, including two samples as DBT adulterated with PBG, and one sample as DBT adulterated with CBG.

**FIGURE 5:**
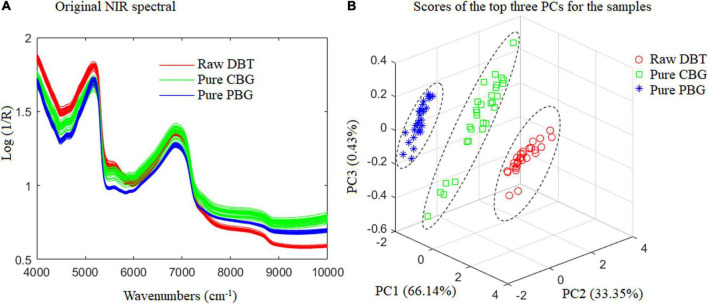
The raw NIR spectral and scatter diagram of the top three principal component analysis scores of the raw duck blood tofu (DBT), pure cow blood-based gel (CBG), and pig blood-based gel (PBG).

The present work used FT-NIR with a spectral range of 4,000–10,000 cm^–1^ for quality analysis of animal blood food. There are differences in basic organic chemicals among the raw DBT, CBG, and PBG used (Table 2). Variations in radiation absorption at different wavenumbers are related to the chemical compositions of the animal blood food (5). As depicted in Figure 5A, each species of animal blood-based food has a characteristic spectrum that allows its identification and differentiation (Figure 5B). This is necessary to detect the DBT binary and ternary adulterated with cow and/or pig blood-based gel using the FT-NIR technique.

**FIGURE 6:**
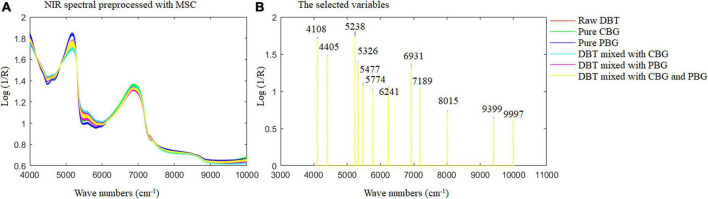
The multivariate scattering correction spectra of the samples prepared and used **(A)** and the selected wavenumbers **(B)** for Fisher LDA and ELM modeling. DBT, Duck blood tofu; CBG, cow blood-based gel; PBG, pig blood gel.

Figure 6 shows the MSC spectra (A) of the samples prepared and used, and the selected wavenumbers (B) for Fisher LDA and ELM modeling. It could be observed from the spectra profile in Figure 6B that the crucial wavenumber for identification of the adulterated DBT samples are around 4,108, 4,405, 5,238, 5,326, 5,477, 5,774, 6,241, 6,931, 7,189, 8,015, 9,399, and 9,997 cm^–1^. These key wavenumbers reflected the difference of hydrogen components in the raw DBT, pure cow, and pig blood-based gels, such as 4,108 and 4,405 cm^–1^ corresponds to C-H+C-C and C-H+C-H combinations, which represent CH, CH_2_, and CH_3_ groups, respectively; 5,238 and 5,326 cm^–1^ represents RCO_2_H and C = O stretch 2nd overtone, respectively; 5,477 cm^–1^ represents C = O stretch 2nd overtone; 5,774 cm^–1^ corresponds to S-H and C-H 1st overtone, which represents S-H, C-H, and C-H_2_; 6,241 cm^–1^ represents Ar-CH; 6,931 cm^–1^ represents ROH, CONH_2_, and CH; 7,189 and 8,015 cm^–1^ corresponds to the second overtone region, which represents CH_3_, CH_2_, ArOH, and CH; 9,399 and 9,997 cm^–1^ corresponds to N-H 2nd overtone, which represents RNH_2_. All groups are associated with significant amounts of protein, amino acids, fat, and sugar is shown in Table 2.

Extreme learning machine performed better than Fisher LDA for the identification of adulterated DBT samples. The reason is primarily due to the relationships among the FT-NIR data matrices of the animal blood food used were more complex than linear as a result of the essential characteristic of FT-NIR, which is spectral of several wavenumbers may contain information from the same organic chemicals, and spectral of each wavenumber may contain chemical information from several organic chemicals in the food materials. ELM has a significant advantage over linear discriminant analysis algorithms for processing non-linear problems due to its superior ability to self-learning and self-adjusting (31).

### Prediction of the adulteration levels

In this section, extreme learning machine regression (ELMR) models were constructed and optimized for predicting adulteration levels. Three FT-NIR datasets of those adulterated samples, namely DBT mixed with CBG, DBT mixed with PBG, and DBT ternary mixed with CBG and PBG were utilized to build four ELMR models. ELMR No. 1 for prediction of the CBG content in DBT adulterated with CBG; ELMR No. 2 for prediction of the PBG content in DBT adulterated with PBG; and ELMR Nos. 3 and 4 for prediction of the CBG and PBG content in the DBT ternary mixed with cow and pig blood-based gels, respectively.

The 1st Der, 2nd Der, centralization, SNV, and MSC separately paired with CARS for spectra denoising and screening of the crucial spectral wavenumber during ELMR modeling according to four convergence criteria: *RMSE*, correlation coefficients (*r*), *RPD*, and *RER*. The selected variables coupled with spectral preprocess technology for ELMR Nos. 1 and 2 were also utilized to construct ELMR Nos. 3 and 4, respectively, but performances were compared and optimized. Therefore, the optimized variables and spectral pretreatment method for ELMR No. 1 may be different from those for ELMR No. 3 because the original FT-NIR datasets were different. The same situation applies to ELMR Nos. 2 and 4.

The Kennard–Stone algorithm was also used to select one-third of samples from each adulteration level group as the prediction set and the remaining samples were utilized as the training set. The Sine activation function (Eq. 10) was used as the activation function of the hidden layers according to the optimized ELM model for identifying adulterated animal blood foods.

The number of hidden neurons was also optimized during ELMR modeling. Herein, as the strategy of cut-and-trial was used, the optimal number of hidden neurons was also set at a range of [1, 50].

**FIGURE 7:**
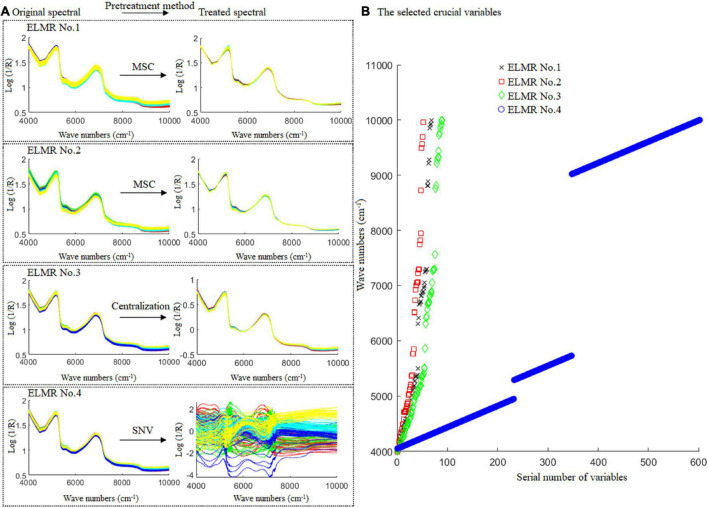
The best pre-treatment technique and the crucial variables selected for the optimal ELMR models constructed. ELMR No. 1 for prediction of the CBG content in DBT adulterated with CBG; ELMR No. 2 for prediction of the PBG content in DBT adulterated with PBG; and ELMR Nos. 3 and 4 for prediction of the CBG and PBG content in the DBT ternary mixed with cow and pig blood-based gels, respectively.

Results show that, for the prediction of CBG content in the DBT adulterated with CBG, the optimal ELMR model was obtained while the MSC and 68 crucial variables were used; for the prediction of PBG content in the DBT adulterated with PBG, the optimal ELMR model was obtained, while the MSC and 52 crucial variables were used; for the prediction of the CBG content in the mixtures of DBT ternary adulterated with CBG and PBG, the optimal ELMR model was obtained while the centralization and 90 crucial variables were used; and for prediction of the PBG content in the mixtures of DBT ternary adulterated with CBG and PBG, the optimal ELMR model obtained while SNV and 602 crucial variables were used. The optimal spectral pretreatment method and the selected crucial variables for constructing the ELMR models are shown in Figure 7.

**FIGURE 8:**
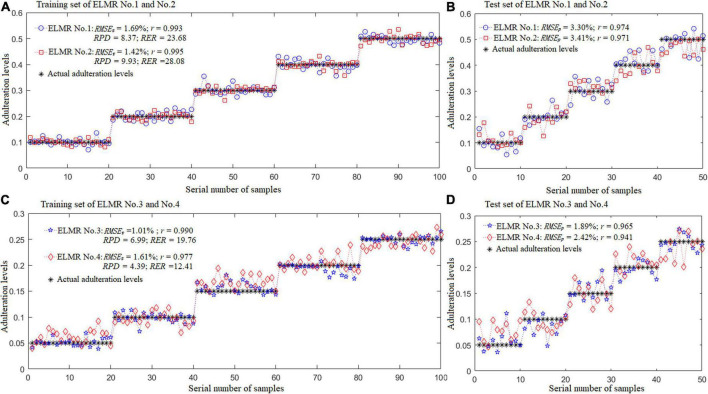
Results of the optimal ELMR models for predicting adulteration levels. **(A,B)** ELMR No. 1 for prediction of the CBG content in DBT adulterated with CBG; ELMR No. 2 for prediction of the PBG content in DBT adulterated with PBG; and **(C,D)** ELMR Nos. 3 and 4 for prediction of the CBG and PBG content in the DBT ternary mixed with cow and pig blood-based gels, respectively.

Figure 8 depicts the actual and predicted adulteration levels of the training and test set of the optimal ELMR models built. As demonstrated in the figure, all the correlation coefficients of the training and prediction set of the optimal ELMR models were higher than 0.94, with the *RMSE*_*t*_ and *RMSE*_*p*_ less than 3.5%, and the *RPD* and *RER* were over 4.0 and 12.0, respectively. Performances of the ELMR models constructed were satisfactory.

## Conclusion

Due to the significant economic importance of DBT, the practice of adulterating or replacing it with other low-cost animal types of blood is still quite severe. The use of FT-NIR in combination with fast chemometrics was presented as a method for detecting DBT binary and ternary adulterated with cow and pig blood-based gels. The ELM model outperformed the Fisher LDA model in identifying adulterated DBT, with higher identification rates of over 93.0% in both the training and test sets. For adulterated level prediction, all the correlation coefficients of the training and prediction sets of the ELMR models constructed were higher than 0.94, with the *RMSE*_*t*_ and *RMSE*_*p*_ all less than 3.5%, and the *RPD* and *RER* were all over 4.0 and 12.0, respectively. The findings of this work show that FT-NIR paired with ELM may be utilized to assess edible animal blood food adulteration rapidly and conveniently.

## Data availability statement

The original contributions presented in this study are included in the article/supplementary material, further inquiries can be directed to the corresponding author.

## Author contributions

FH conceived and designed the FT-NIR experiments, chemicals measurements, and data modeling. LM and XZ performed the measurements. FH, LM, JA, and MR wrote and revised the manuscript. FH and XH supervised the execution of analyses. All authors contributed to the article and approved the submitted version.
